# Architects and Bath Rooms

**Published:** 1856-04

**Authors:** 


### ARCHITECTS AND BATH ROOMS.

WE do not believe there is one Architect in the United States known to favorable fame who is capable of doing either one of two things, viz.:

Of erecting a public building that will not leak, or,

Of constructing a dwelling heated by a furnace which cannot take fire.

Perhaps they are not to blame—the odium may be due to parsimonious landlords. But wherever the fault is chargeable, intelligent and observing men know very well that there is scarcely a public building in New-York of late erection which has not leaked, or which does not now leak. And who knows a perfectly safe flue in all New York? Is there an Architect who understands the philosophy of a Bath-Room? The room of all others, except a permanent sitting room, which most needs a flue or register, or other heating apparatus attached to it, is the "Bath Room," and yet we have never seen a bath room with such an attachment.

Let us for a moment lay aside all book and newspaper knowledge, all preconceived notions, and consult our feelings in the operation of that kind of bathing whose object is to make the body clean of the grease, scales, dust, &c., which are constantly accumulating on its surface.

We all know that cold water will not make the hands clean, nor will hard water, even if it is warm. Hence when we wish to wash ourselves very clean, we use warm water with soap, and if we can get it, rain or cistern, or snow water.

With the present habits of civilized life, comparatively few persons among the middle and upper classes of society have vitality enough to make a cold bath advisable: they have not that "reaction" which gives to a cold bath its highest advantage, hence even the most rabid cold-waterist does not advise cold baths under such circumstances. Then when we take into account how many children there are who are too young for a cold bath, that old have not the stamina for it, whilst to that large number, neither infants, nor aged nor young people, but "children" who roll about in the dust, and mud, and snow, who sprawl upon the floor and dabble in water and dirt, the bathing which is most needed, is a cleansing operation; nothing short of soap, warm water, and a bristle brush will meet their demands, so that after all, especially in winter time, by nine persons out of ten in the whole community of those who practise bathing, the tepid or warm bath is what is needed. In fact, only the very small class of persons who are robust "can stand" a cold bath in winter, and in our opinion such persons do not need it. *If you are well, let yourself alone as to remedial means, for you can't be better than well.* Personally, this is our theory and practice too, we never had a cold bath but once, since boyhood, and that made us sick, and we shudder at the thought of a cold bath ever since. We believe cold shower baths are the ordinary punishments inflicted on refractory convicts in our penitentiaries, we have understood that they regard them with the greatest dread, and yet there are wiseacres among us who daily submit themselves to that self-infliction. Such persons have expatiated in eloquent terms as to the delightful feelings experienced after the operation is over of a morning. As for that matter, we feel delightful of a morning without all that trouble and penance. The perusal of the morning papers before a bright coal fire in the grate makes us feel delightful, and more delightful still, hearing now and then the meanwhile, the rapid patting of the little feet of our children on the floor above us, as they get out of bed, and run to the fire, being the first telegraphic message to us in the morning that they have waked up well, merry and happy. This is a feeling more purely delightful than any cold shower bath can originate, without the preceding "shock" which we always think of with a shudder. So our advice is, if you want to feel "delightful" of a winter's morning, have a young dozen of little children about the house *your own*, *mind*—take one or two morning papers, and pay for them in advance, (for there's a singular virtue in that,) get up, dress for the day, and be seated before a brisk, burning fire by the time it is fairly light enough to read, be sure tho' to have no "bills payable" that day, for that will spoil all the fun. There's nothing "delightful" under such circumstances, when there are no assets to draw upon.

But we have unconsciously wandered from the bath-room to Wall-street. Surely we are getting worldly-minded. The fact is, we have not felt as great an interest in our Journal of late as we used to. Wonder if our readers have been led to surmise the same thing! Ah me, we find ourselves, and most unwillingly, arriving at the experience of Lord Byron, when he declared that he had come to the point of his life in which he began to feel the highest possible respect for the smallest amount of current coin, and we find within us a growing, loving attraction towards the avocations which—as Jonathan would say, "*pay best!*" Our experience and observation convince us that nine men out of ten will pay in experiments for regaining health a thousand dollars, more cheerfully than they would pay one for information, which, if acted upon, would certainly preserve it, and fortunate it is for us Doctors that the masses are such numskulls, else we would find our occupation gone, and would have to go to cracking rocks for the turnpike, or picking oakum.

When we sat down we intended to tell on one side of a half-sheet, how a bath-room ought to be constructed, but our mind is forever calculating how much money this six inches of snow on the morning of the nineteenth day of March, Anno Domini, eighteen hundred and fifty-six, with zero just ten days ago, will bring us—hope it will be "considerable" any how. Now for the bath-room, desperately.

As nine-tenths, if not its whole use in winter, at least in the majority of families, is for cleansing purposes, the water should be warm, but if the body emerges from the water into an atmosphere much colder, we all know the uncomfortableness of the feeling which follows; this causes us to perform the operation hurriedly and consequently slightingly, while to a great many the more serious result is a severe cold, causing days and weeks, if not months of subsequent discomfort or illness. The water then in the bath-room should feel *comfortably warm*, while the air of the room should be of such a temperature, as to prevent any sensation of coldness amounting to the disagreeable. It is safer to be guided by one's feelings as to the temperature of the water and the air of the room than a thermometer; for not only do different persons, by reason of their different degrees of health require a different temperature, but for the same reason, the same person may require a temperature to-day, which would not be suitable a week or a month hence.

